# Treatment rates and healthcare costs of patients with fragility fracture by site of care: a real-world data analysis

**DOI:** 10.1007/s11657-023-01229-7

**Published:** 2023-03-11

**Authors:** A. Singer, M. R. McClung, O. Tran, C. D. Morrow, S. Goldstein, R. Kagan, M. McDermott, A. Yehoshua

**Affiliations:** 1 MedStar Georgetown University Hospital, Washington, DC USA; 2 Georgetown University Medical Center, Washington, DC USA; 3 Oregon Osteoporosis Center, Portland, OR USA; 4 Mary MacKillop Institute for Health Research, Australian Catholic University, Melbourne, VIC Australia; 5 Merative, Cambridge, MA USA; 6 NYU Grossman School of Medicine, New York, NY USA; 7 University of California, San Francisco, CA USA; 8 Amgen Inc, Thousand Oaks, CA USA

**Keywords:** Aged, Bone density/drug effects, Bone density conservation agents, Cost of illness, Female, Health care costs, Hospitalization, Humans, Medicare, Middle aged, Postmenopausal, Risk assessment, USA/epidemiology

## Abstract

***Summary*:**

In a characterization of treatment rates and healthcare costs among patients with an osteoporotic-related fragility fracture overall and by site of care, costs were high and treatment rates were low.

**Purpose:**

Osteoporotic fractures can be debilitating, even fatal, among older adults. The cost of osteoporosis and related fractures is projected to increase to more than $25 billion by 2025. The objective of this analysis is to characterize disease-related treatment rates and healthcare costs of patients with an osteoporotic fragility fracture overall and by site of fracture diagnosis.

**Methods:**

In this retrospective analysis, individuals with fragility fractures were identified in the Merative MarketScan® Commercial and Medicare Databases among women 50 years of age or older and diagnosed with fragility fracture between 1/1/2013 and 6/30/2018 (earliest fracture diagnosis = index). Cohorts were categorized by clinical site of care where the diagnosis of fragility fracture was made and were continuously followed for 12 months prior to and following index. Sites of care were inpatient admission, outpatient office, outpatient hospital, emergency room hospital, and urgent care.

**Results:**

Of the 108,965 eligible patients with fragility fracture (mean age 68.8), most were diagnosed during an inpatient admission or outpatient office visit (42.7%, 31.9%). The mean annual healthcare costs among patients with fragility fracture were $44,311 (± $67,427) and were highest for those diagnosed in an inpatient setting ($71,561 ± $84,072). Compared with other sites of care at fracture diagnosis, patients diagnosed during an inpatient admission also had highest proportion of subsequent fractures (33.2%), osteoporosis diagnosis (27.7%), and osteoporosis therapy (17.2%) during follow-up.

**Conclusion:**

The site of care for diagnosis of fragility fracture affects treatment rates and healthcare costs. Further studies are needed to determine how attitude or knowledge about osteoporosis treatment or healthcare experiences differ at various clinical sites of care in the medical management of osteoporosis.

## Background

Osteoporosis is a skeletal disease characterized by the loss of bone mass and the deterioration of bone microarchitecture, wherein bone strength is compromised and affected patients are predisposed to an elevated risk of fracture [1]. These fractures, also known as fragility fractures, typically occur in wrists, hips, and vertebrae, can often be debilitating, put patients at an increased risk for a subsequent fracture, and can even be fatal among older adults [2]. Globally, women over the age of 50 have a 9.8 to 22.8% lifetime risk of fragility fractures and fractures will occur among 1 in 3 [3]. The Women’s Health Initiative Observational study projected the number of fractures as similar to or higher than breast cancer, stroke, and cardiovascular disease events combined among women aged 50–79 in the USA [4]. The Bone Health and Osteoporosis Foundation (BHOF; formerly the National Osteoporosis Foundation) estimates 3 million fractures and $25.3 billion in direct healthcare costs per year by 2025 [5].

Due to undertreatment and disease mismanagement, osteoporosis and related fractures present a substantial cost burden to the healthcare system. Osteoporotic fracture is a top driver of hospitalization-related costs among US women—more costly than breast cancer, myocardial infarction, and stroke [6]. One study estimated the national cost of osteoporosis and related fractures to be $22 billion [7], and that cost is expected to escalate to more than $95 billion by 2040 [8].

Fracture prevention and earlier osteoporosis diagnosis are essential to initiation of adequate treatment; however, osteoporosis remains underdiagnosed among fragility fracture patients [9]. Frequency of osteoporosis diagnosis varies by site of care, and we hypothesize diagnosis patterns similarly differ across provider specialty type [10]. Undertreatment is due in part to underdiagnosis among these patients [11]. Bisphosphonates have been widely used to treat bone diseases since the 1970s and are well established as the first-line treatment for osteoporosis. However, poor adherence is common with oral bisphosphonates. Non-persistent patients remain at elevated risk for fracture [12]. Low persistence is due in part to complex dosing instructions and fear of side effects [6, 12, 13].

Osteoporosis is treated by a range of clinicians in a variety of settings [14]. Although there is a high degree of consistency and agreement regarding osteoporosis treatment guidelines, recommendations, and practice among clinicians, there are also significant differences. For example, the American College of Physicians recommends against bone density monitoring during the 5-year pharmacologic treatment period for osteoporosis in women, whereas the American Association of Clinical Endocrinologists recommend bone density monitoring every 1–2 years [15, 16]. Currently, there is a lack of research describing the relationship between the site of care where a patient is diagnosed with a fragility fracture with healthcare resource utilization, healthcare costs, osteoporosis diagnosis, osteoporosis treatment patterns, and subsequent fragility fracture rates in the following year.

## Objective

To characterize baseline demographic characteristics and clinical conditions and the 12-month patient journey following a fragility fracture. Treatment rates and healthcare costs of individuals with fragility fracture were reported by the site of care where they were diagnosed.

## Methods

### Study design and data source

This observational cohort study was conducted using de-identified data from the Merative MarketScan® Commercial Claims Database and the Medicare Supplemental and Coordination of Benefits Database. The commercial claims database contains the inpatient, outpatient, and prescription drug experience of employees and their dependents, covered under a variety of fee-for-service and managed care health plans, including approximately 89 million lives from 2012 to 2018. The Medicare database contains the healthcare experience of retirees with Medicare supplemental insurance paid for by employers, including 5.5 million lives between 2012 and 2018. These databases provided detailed cost, use, and outcomes data for healthcare services performed in both inpatient and outpatient settings. Data were extracted using International Classification of Diseases, 9th and 10th Revision, Clinical Modification (ICD-9-CM and ICD-10-CM) codes, Current Procedural Terminology (CPT) 4th edition codes, Healthcare Common Procedure Coding System (HCPCS) codes, and National Drug Codes (NDCs). These de-identified data were fully compliant with US patient confidentiality requirements set forth in the Health Insurance Portability and Accountability Act of 1996.

### Patient selection and site of care cohort assignment

Women aged 50 years of age and older with a fragility fracture (index date = date of diagnosis of first fracture) were identified in the commercial and Medicare Databases during January 1, 2013 through June 30, 2018. Fragility fracture, osteoporosis, and other clinical conditions were identified by ICD-9-CM/ICD-10-CM diagnosis or CPT procedure coding. To determine eligibility, patients had at least 12 months of continuous enrollment and pharmacy benefits prior to the index date (baseline period) and at least 12 months of continuous enrollment and pharmacy benefits following the index date (follow-up period). Patients with Paget’s disease of the bone, osteitis deformans, osteogenesis imperfecta, hypercalcemia, cancer, or conditions categorized in ICD-9-CM/ICD-10-CM as “other osteopathy” during the baseline were excluded.

Individuals with fragility fracture were categorized into cohorts based on the site of care at diagnosis. Sites of care of interest were identified a priori by the co-authors who treat and study osteoporosis and fragility fracture: inpatient, outpatient office, outpatient hospital, emergency room (ER), federally qualified health center (FQHC), rehabilitation facility, nursing facility, urgent care, patient home, rural health clinic, and assisted living facility. Detailed data was not reported for cohorts with less than 30 individuals.

Individuals were also categorized into cohorts based on the index physician specialty. The index physician specialty was the physician specialty that made the diagnosis of fragility fracture. Specialties of interest on the first fragility fracture diagnosis were family medicine, internal medicine, obstetrics/gynecology (OB-GYN), orthopedics, geriatrics, rheumatology, endocrinology, and emergency medicine.

### Patient characteristics

Patient demographic characteristics included age, region, and health plan measured on index date. Clinical characteristics, including the Deyo-Charlson Comorbidity Index (DCI)[17], were reported during the 12-month baseline period.

### Outcomes

All-cause and disease-related healthcare utilization and costs were measured during the 12-month follow-up period. The index date was included in the follow-up period; therefore, healthcare utilization and costs of the index event are captured in the post-index averages of services and treatment. Disease-related healthcare utilization and costs corresponded to medical claims with a diagnosis code for osteoporosis or osteopenia, a diagnosis or procedure code for fragility fracture (defined by the previously described algorithm [18]), medical claims with administration (HCPCS codes) for osteoporosis therapies, or outpatient pharmacy claims (NDC codes) for osteoporosis therapies. This study reports all-cause and disease-related healthcare utilization and costs for inpatient, ER, and outpatient health care settings, as well as pharmacy utilization and costs.

Healthcare costs are reported in 2018 constant US dollars, adjusted using the Medical Care component of the Consumer Price Index. Healthcare costs were measured using the financial fields on administrative claims in the MarketScan Databases.

Proportions of patients with any osteoporosis treatment during the follow-up period were reported. Osteoporosis treatments covering multiple classes of anti-resorptive and bone forming agents included denosumab (RANKL inhibitor), alendronate (bisphosphonate), ibandronate (bisphosphonate), risedronate (bisphosphonate), zoledronate (bisphosphonate), raloxifene (selective estrogen receptor modulator), and teriparatide (parathyroid hormone analog) and were measured in the 12-month follow-up period. The time to subsequent fracture was measured as the number of days from the index date to the earliest fracture diagnosis during the follow-up period. Anatomical site of fracture was defined by diagnosis of a fragility fracture during the 12-month follow-up period and type of fracture (hip, vertebra, and non-hip non-vertebral) was also reported. Repeat fractures defined as those that occurred more than 90 days following the index fracture of the identical fracture type were also reported.

Bone density scans were measured in the baseline and follow-up periods. Bone density scans included procedure codes that describe dual-energy X-ray absorptiometry (DEXA), bone density studies on one or more sites, ultrasound bone density measurement and interpretation, and single energy x-ray absorptiometry (SEXA) bone density studies.

### Statistical analysis

Mean and standard deviation (SD) were reported for continuous variables, while frequencies and percentages were reported for categorical variables. All data analyses were conducted using WPS version 4.1 (World Programming, UK).

## Results

### Study population

**Fig. 1:**
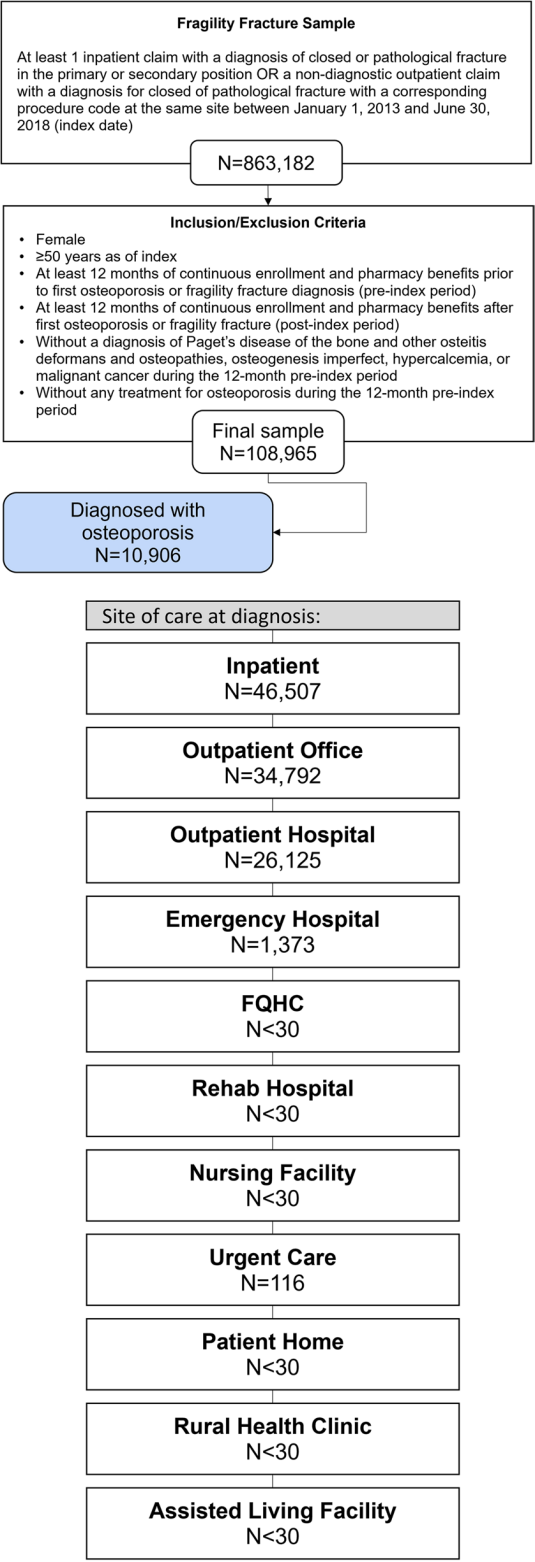
Patient selection. Abbreviations: *FQHC*, Federally Qualified Health Center

Of the 108,965 eligible patients with fragility fracture, most were diagnosed during an inpatient admission, outpatient office visit, or outpatient hospital visit (42.7%, 31.9%, 24.0%; Fig. 1). All other sites of care identified less than 2% of the fragility fracture groups. The largest cohort of patients with fragility fracture was aged between 50–64 (48.8%), with an average age of 68.8 years (Table 1). Patients with fragility fracture diagnosed during an inpatient admission were older on average (75.0 years) compared with the overall group (68.8 years); meanwhile, most patients indexed in all other settings were between ages 50 and 64. Most patients had an Exclusive Provider Organization (EPO) health insurance plan (49.9%). The largest proportion of patients originated from the South (35.4%). Ten percent of the fragility fracture cohort had a diagnosis of osteoporosis during baseline (Fig. 1). Average baseline all-cause healthcare costs were $18,146 (SD $45,537; Table 1). The mean Deyo-Charlson comorbidity index score was 0.9 (SD 1.4), and the most common comorbidities included hypertension (52.9%), dyslipidemia (40.0%), and respiratory diseases (36.6%; Table 1).

**Table 1:** Demographics and clinical characteristics among patients with fragility fracture (overall and stratified by site of care at diagnosis)

	All patients	Inpatient admission	Outpatient office	Outpatient hospital	Emergency room hospital	Urgent care
	N/Mean (%/SD)	N/Mean (%/SD)	N/Mean (%/SD)	N/Mean (%/SD)	N/Mean (%/SD)	N/Mean (%/SD)
	*N* = 108,965	*N* = 46,507	*N* = 34,792	*N* = 26,125	*N* = 1373	*N* = 116
	(100.0%)	(42.7%)	(31.9%)	(24.0%)	(1.3%)	(0.1%)
Age	68.8 (12.8)	75.0 (12.8)	64.7 (11.1)	63.8 (10.5)	61.6 (9.5)	61.2 (9.1)
Age group						
50–64	53,125 (48.8%)	13,017 (28.0%)	21,616 (62.1%)	17,305 (66.2%)	1072 (78.1%)	85 (73.3%)
65–74	17,722 (16.3%)	7379 (15.9%)	5971 (17.2%)	4204 (16.1%)	137 (10.0%)	21 (18.1%)
75–84	20,433 (18.8%)	12,704 (27.3%)	4546 (13.1%)	3062 (11.7%)	106 (7.7%)	8 (6.9%)
85 +	17,685 (16.2%)	13,407 (28.8%)	2659 (7.6%)	1554 (5.9%)	58 (4.2%)	2 (1.7%)
Insurance plan type						
Comprehensive	29,252 (26.8%)	17,235 (37.1%)	6825 (19.6%)	5104 (19.5%)	68 (5.0%)	11 (9.5%)
EPO/PPO	54,392 (49.9%)	20,528 (44.1%)	19,547 (56.2%)	13,481 (51.6%)	754 (54.9%)	59 (50.9%)
POS with Capitation	562 (0.5%)	275 (0.6%)	156 (0.4%)	124 (0.5%)	7 (0.5%)	0 (0.0%)
HMO	8979 (8.2%)	3529 (7.6%)	2550 (7.3%)	2527 (9.7%)	339 (24.7%)	18 (15.5%)
POS	5905 (5.4%)	2227 (4.8%)	1847 (5.3%)	1791 (6.9%)	32 (2.3%)	8 (6.9%)
Other	8578 (7.9%)	2067 (4.4%)	3516 (10.1%)	2819 (10.8%)	153 (11.1%)	19 (16.4%)
Unknown	1297 (1.2%)	646 (1.4%)	351 (1.0%)	279 (1.1%)	20 (1.5%)	1 (0.9%)
Payer						
Commercial	52,281 (48.0%)	12,662 (27.2%)	21,316 (61.3%)	17,118 (65.5%)	1070 (77.9%)	85 (73.3%)
Medicare	56,684 (52.0%)	33,845 (72.8%)	13,476 (38.7%)	9007 (34.5%)	303 (22.1%)	31 (26.7%)
Geographic region						
Northeast	24,828 (22.8%)	10,194 (21.9%)	10,022 (28.8%)	4372 (16.7%)	205 (14.9%)	N < 30
North Central	33,375 (30.6%)	14,684 (31.6%)	10,298 (29.6%)	7983 (30.6%)	347 (25.3%)	50 (43.1%)
South	38,626 (35.4%)	15,993 (34.4%)	11,958 (34.4%)	9997 (38.3%)	642 (46.8%)	30 (25.9%)
West	11,712 (10.7%)	5505 (11.8%)	2352 (6.8%)	3644 (13.9%)	178 (13.0%)	N < 30
Unknown	424 (0.4%)	131 (0.3%)	162 (0.5%)	129 (0.5%)	N < 30	N < 30
Baseline all-cause healthcare costs (Mean, SD)	$18,146 ($45,537)	$23,532 ($59,507)	$14,868 ($31,952)	$13,377 ($29,425)	$9874 ($20,226)	$8262 ($19,766)
Deyo-Charlson Comorbidity Index (DCI) (Mean, SD)	0.9 (1.4)	1.2 (1.7)	0.7 (1.3)	0.6 (1.1)	0.5 (1.1)	0.4 (1.1)
Hypertension	57,691 (52.9%)	29,366 (63.1%)	16,387 (47.1%)	11,324 (43.3%)	547 (39.8%)	39 (33.6%)
Dyslipidemia	43,536 (40.0%)	19,899 (42.8%)	13,640 (39.2%)	9459 (36.2%)	481 (35.0%)	35 (30.2%)
Respiratory diseases	39,897 (36.6%)	16,926 (36.4%)	13,234 (38.0%)	9206 (35.2%)	470 (34.2%)	38 (32.8%)
Cardiovascular disease	30,837 (28.3%)	18,011 (38.7%)	7523 (21.6%)	5065 (19.4%)	213 (15.5%)	13 (11.2%)
Osteoarthritis	22,916 (21.0%)	11,852 (25.5%)	6366 (18.3%)	4492 (17.2%)	185 (13.5%)	10 (8.6%)
Acute respiratory diseases	24,107 (22.1%)	8971 (19.3%)	8763 (25.2%)	6006 (23.0%)	326 (23.7%)	29 (25.0%)
Chronic respiratory diseases	15,833 (14.5%)	7982 (17.2%)	4626 (13.3%)	3069 (11.7%)	133 (9.7%)	12 (10.3%)
Rheumatoid arthritis	3255 (3.0%)	1710 (3.7%)	894 (2.6%)	623 (2.4%)	25 (1.8%)	2 (1.7%)

Abbreviations: *DCI*, Deyo-Charlson Comorbidity Index; *EPO*, Exclusive Provider Organization; *HMO*, Health Maintenance Organization; *POS*, Point of Service; *PPO*, Preferred provider organization; *SD*, Standard Deviation

### Descriptive outcomes

#### Fragility fracture rates, sites of fragility fracture during follow-up, and bone density scan utilization

During the follow-up period, rate of a subsequent fragility fracture was high (26.6%; Table 2). The most common type of fracture during the follow-up period was non-hip non-vertebral (20.2%; Table 2), with a particularly high number of fractures among patients who indexed in inpatient, ER, and urgent care settings (19.7%, 68.3%, and 35.3%, respectively; Table 3). The wrist/radius-ulna site was the most common site among the non-hip non-vertebral fractures. Patients diagnosed during an inpatient admission (*N* = 46,507) were more likely to have a subsequent hip (13.3%) or vertebral fracture (4.5%), compared with those diagnosed at any other site (Table 3).

**Table 2:** Fragility fracture characteristics during baseline, on index, and follow-up periods (overall cohort)

	12-month baseline or on index	12-month follow-up
	*N* (%)	*N* (%)
	*N* = 108,965	*N* = 108,965
Site of fragility fracture; on index and during follow-up (N,%)		
Presence of fragility fracture, any site	108,965 (100.0%)	28,968 (26.6%)
Hip	18,414 (16.9%)	6632 (6.1%)
Vertebra	10,737 (9.9%)	2719 (2.5%)
Non-hip non-vertebra	86,632 (79.5%)	21,966 (20.2%)
Femur	5107 (4.7%)	1651 (1.5%)
Pelvis	4467 (4.1%)	1448 (1.3%)
Clavicle	2365 (2.2%)	545 (0.5%)
Wrist/radius-ulna	35,647 (32.7%)	9043 (8.3%)
Humerus	14,548 (13.4%)	3379 (3.1%)
Tibia-fibular	7105 (6.5%)	1490 (1.4%)
Ankle	19,233 (17.7%)	4196 (3.9%)
Repeat (same site) fracture occurring > 90 days after index fracture) (N, %)		4849 (4.5%)
Physician specialty on index and follow-up care with the same index physician specialty (N, %		
Family practice (N, %)	18,418 (16.9%)	8846 (48.0%)
Internal medicine (N, %)	12,970 (11.9%)	3074 (23.7%)
OB/GYN (N, %)	78 (0.1%)	25 (32.1%)
Orthopedics (N, %)	40,693 (37.3%)	26,587 (65.3%)
Geriatrics (N, %)	47 (< 0.1%)	4 (8.5%)
Rheumatologist (N, %)	22 (< 0.1%)	8 (36.4%)
Endocrinologist (N, %)	44 (< 0.1%)	12 (27.3%)
Emergency medicine (N, %)	12,633 (11.6%)	275 (2.2%)
Follow-up care with physician specialty different than index physician specialty		
Family practice (N, %)		9185 (8.4%)
Internal medicine (N, %)		8897 (8.2%)
OB/GYN (N, %)		2038 (1.9%)
Orthopedics (N, %)		20,624 (18.9%)
Geriatrics (N, %)		171 (0.2%)
Rheumatologist (N, %)		2173 (2.0%)
Endocrinologist (N, %)		1820 (1.7%)
Emergency medicine (N, %)		367 (0.3%)
Number of unique care physician specialties during follow-up (Mean, SD)		0.6 (0.8)
Bone density scan, including DXA; during baseline and follow-up (N, %)	9505 (8.7%)	15,011 (13.8%)

Abbreviations: *DXA*, dual-energy x-ray absorptiometry; *OB/GYN*, obstetrics/gynecology

**Table 3:** Fragility fracture outcomes during the 12-month follow-up period stratified by site of care^1^ at diagnosis

	Inpatient admission	Outpatient office	Outpatient hospital	Emergency room hospital	Urgent care
	N (%)	N (%)	N (%)	N (%)	N (%)
	*N* = 46,507	*N* = 34,792	*N* = 26,125	*N* = 1373	*N* = 116
Site of fragility fracture					
Presence of fragility fracture, any site	15,434 (33.2%)	4275 (12.3%)	8271 (31.7%)	941 (68.5%)	41 (35.3%)
Hip	6175 (13.3%)	264 (0.8%)	188 (0.7%)	3 (0.2%)	0 (0.0%)
Vertebra	2092 (4.5%)	289 (0.8%)	333 (1.3%)	5 (0.4%)	0 (0.0%)
Non-hip non-vertebral	9145 (19.7%)	3909 (11.2%)	7928 (30.3%)	938 (68.3%)	41 (35.3%)
Femur	1501 (3.2%)	89 (0.3%)	57 (0.2%)	3 (0.2%)	0 (0.0%)
Pelvis	1271 (2.7%)	105 (0.3%)	69 (0.3%)	3 (0.2%)	0 (0.0%)
Clavicle	197 (0.4%)	88 (0.3%)	231 (0.9%)	27 (2.0%)	1 (0.9%)
Wrist/Radius-Ulna	1443 (3.1%)	2044 (5.9%)	4907 (18.8%)	623 (45.4%)	25 (21.6%)
Humerus	1456 (3.1%)	752 (2.2%)	1064 (4.1%)	101 (7.4%)	6 (5.2%)
Tibia-Fibular	953 (2.0%)	218 (0.6%)	287 (1.1%)	30 (2.2%)	2 (1.7%)
Ankle	1582 (3.4%)	767 (2.2%)	1636 (6.3%)	201 (14.6%)	8 (6.9%)

^1^Site of care where N < 30 not reported

#### Diagnosis and treatment of osteoporosis

To understand the patient journey after diagnosis of fragility fracture, physician specialty at time of diagnosis, and the physician specialty for subsequent care are reported in Table 2. Approximately 20% of all individuals with fragility fracture (*N* = 108,965) were diagnosed with osteoporosis during the follow-up period and the rate was notably higher among those diagnosed in an inpatient setting, 27.7% compared with other sites of care (9.5–15.7%) (Table 4). Patients whose index fragility fracture was diagnosed in an inpatient setting also had the highest proportion of osteoporosis therapy during follow-up (17.2% vs 8.6–12.9% in outpatient settings). Among the subset of all fragility fracture patients treated with osteoporosis therapy during follow-up (*N* = 15,342), most were treated with oral bisphosphonates (alendronate 45.6%, ibandronate 11.2%, risendronate 7.6%) despite being a high-risk group for a subsequent fracture (Table 4). Among patients with osteoporosis treatment, the proportion of days covered (during the 12-month follow-up period) was 51.9% and the mean time from index date to therapy initiation was 109 ± 0.2 days (and generally consistent across settings with the exception of urgent care where time to treatment was only 48 ± 0.2 days; Table 4). Among individuals who utilized denosumab for osteoporosis treatment (*N* = 2,564), their mean time to treatment initiation was lengthier at 157 ± 94.7 days. The lowest treatment rates occurred among patients in the urgent care, outpatient office, and ER hospital cohorts (8.6%, 10.9%, and 10.9%, respectively; Table 4).

**Table 4:** Osteoporosis therapy measured during the 12-month follow-up period; overall and stratified by site of care at fragility fracture diagnosis

	All patients	Inpatient admission	Outpatient office	Outpatient hospital	Emergency room hospital	Urgent care
	N/Mean (%/SD)	N/Mean (%/SD)	N/Mean (%/SD)	N/Mean (%/SD)	N/Mean (%/SD)	N/Mean (%/SD)
	*N* = 108,965	*N* = 46,507	*N* = 34,792	*N* = 26,125	*N* = 1373	*N* = 116
Patients with an osteoporosis diagnosis (N,%) during the follow-up period	21,339 (19.6%)	12,887 (27.7%)	4168 (12.0%)	4090 (15.7%)	176 (12.8%)	11 (9.5%)
Total number of patients with any osteoporosis therapy during the follow-up period (N, %)^1^	15,342 (14.1%)	8007 (17.2%)	3797 (10.9%)	3372 (12.9%)	150 (10.9%)	10 (8.6%)
RANK ligand inhibitor						
denosumab	2564 (16.7%)	1412 (17.6%)	596 (15.7%)	530 (15.7%)	25 (16.7%)	1 (10.0%)
Bisphosphonates						
Alendronate	6990 (45.6%)	3652 (45.6%)	1734 (45.7%)	1516 (45.0%)	81 (54.0%)	5 (50.0%)
Ibandronate	1718 (11.2%)	826 (10.3%)	431 (11.4%)	442 (13.1%)	18 (12.0%)	1 (10.0%)
Risedronate	1173 (7.6%)	565 (7.1%)	326 (8.6%)	276 (8.2%)	3 (2.0%)	2 (20.0%)
Zoledronate	1139 (7.4%)	606 (7.6%)	266 (7.0%)	263 (7.8%)	3 (2.0%)	0 (0.0%)
Selective estrogen receptor modulators						
Raloxifene	1547 (10.1%)	727 (9.1%)	447 (11.8%)	349 (10.3%)	20 (13.3%)	2 (20.0%)
Parathyroid hormone analogues						
Teriparatide	1207 (7.9%)	765 (9.6%)	201 (5.3%)	231 (6.9%)	10 (6.7%)	0 (0.0%)
Time to (days) treatment initiation (Mean, SD)						
Any osteoporosis therapy^2^	109 (0.2)	112 (0.2)	102 (0.2)	109 (0.2)	106 (0.2)	48 (0.2)
denosumab	157 (94.7)	165 (94.0)	146 (94.2)	146 (94.9)	169 (91.7)	110 (0.0)
Proportion of days covered (PDC)^3^ with any osteoporosis therapy over the follow-up period (Mean, SD)	51.9% (28.1%)	50.5% (27.3%)	53.5% (28.9%)	53.5% (28.7%)	52.3% (29.5%)	59.3% (31.4%)

^1^The percentages calculated for the subsequent rows are calculated out of the total number of patients with any osteoporosis therapy

^2^Any osteoporosis therapy includes the following drugs: alendronate, denosumab, ibandronate, raloxifene, risedronate, teriparatide, zoledronic acid

^3^PDC is defined as the number of days covered by the reported days’ supply of a pharmacy claim or the days of clinical benefit of an outpatient medical claim, divided by 365 days

Of the 108,965 individuals with fragility fracture, most (37.3%) were diagnosed by an orthopedist on the index date, followed by a family practice physician (16.9%; Table 2). Similarly, subsequent care from the index physician specialty was most common among the orthopedists (65.3%) and family practice physicians (48.0%). When subsequent care was obtained from a different physician specialty from the index provider, orthopedists were the most common specialist (18.9%). Among index physician specialty, patients whose index fragility fracture diagnosis was made by rheumatologists and geriatricians had the highest osteoporosis treatment rates (31.8% and 23.4%, respectively), while patients whose index fragility fracture diagnosis was made by orthopedics had the lowest treatment rate (11.7%).

#### Healthcare utilization and costs

**Fig. 2:**
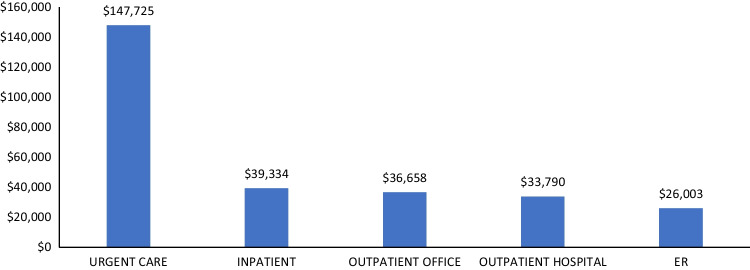
Inpatient admission costs measured during the 12-month follow-up period stratified by site of care at fragility fracture diagnosis. Abbreviation: *ER*, Emergency room

**Fig. 3:**
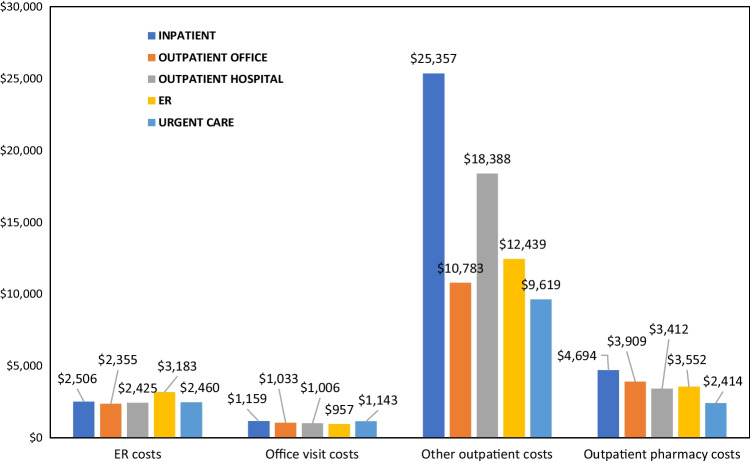
Outpatient costs measured during the 12-month follow-up period stratified by site of care at fragility fracture diagnosis. Abbreviation: *ER*, Emergency room

The mean annual healthcare costs among patients with fragility fracture were $44,311 (± $67,427). Annual healthcare costs were highest for those diagnosed in an inpatient setting ($71,561 ± $84,072; Table 5). Among patients with at least one inpatient admission, hospitalization costs were lowest for patients with fragility fracture diagnosed in an ER ($26,003 ± $29,304) and highest for those diagnosed at urgent care ($147,725 ± $323,368) (Fig. 2). Outpatient costs (office visits) were generally lowest for those diagnosed at urgent care and highest for those diagnosed in an inpatient setting (Fig. 3).

**Table 5:** All-cause & disease-related healthcare utilization and expenditures during the 12-month follow-up period; overall and stratified by site of care at fragility fracture diagnosis

	All patients	Inpatient admission	Outpatient office	Outpatient hospital	Emergency room hospital	Urgent care
	N/Mean (%/SD)	N/Mean (%/SD)	N/Mean (%/SD)	N/Mean (%/SD)	N/Mean (%/SD)	N/Mean (%/SD)
	*N* = 108,965	*N* = 46,507	*N* = 34,792	*N* = 26,125	*N* = 1373	*N* = 116
Total all-cause healthcare costs	$44,311 ($67,427)	$71,561 ($84,072)	$20,867 ($41,874)	$28,236 ($40,190)	$23,193 ($27,846)	$26,380 ($103,122)
Total costs of outpatient prescriptions	$4121 ($11,637)	$4694 ($11,275)	$3909 ($13,817)	$3412 ($8780)	$3552 ($11,349)	$2414 ($5399)
Emergency room (ER) visits						
Patients with an ER visit	49,946 (45.8%)	24,052 (51.7%)	10,014 (28.8%)	14,475 (55.4%)	1373 (100.0%)	27 (23.3%)
Number of ER visits	1.8 (1.8)	2.1 (2.0)	1.7 (1.5)	1.6 (1.4)	1.7 (2.4)	1.6 (1.1)
Total ER costs	$2471 ($5705)	$2506 ($6836)	$2355 ($5054)	$2425 ($3785)	$3171 ($5174)	$2460 ($7112)
Outpatient office visits						
Patients with an office visit	104,081 (95.5%)	43,186 (92.9%)	34,792 (100.0%)	25,324 (96.9%)	1332 (97.0%)	116 (100.0%)
Number of office visits	10.0 (8.1)	10.8 (8.6)	9.4 (7.8)	9.4 (7.5)	9.0 (6.7)	9.6 (6.4)
Total outpatient office visit costs	$1085 ($1008)	$1159 ($1094)	$1033 ($981)	$1006 ($891)	$957 ($746)	$1143 ($813)
Inpatient admissions						
Patients with an admission	50,000 (45.9%)	46,507 (100%)	4428 (12.7%)	3274 (12.5%)	172 (12.5%)	10 (8.6%)
Number of admissions	1.4 (0.9)	1.3 (0.9)	1.3 (0.8)	1.3 (0.8)	1.3 (0.8)	1.2 (0.4)
Average Length of Stay	5.0 (4.8)	4.6 (4.9)	4.5 (4.7)	4.4 (4.4)	4.6 (5.4)	6.3 (8.5)
Total inpatient costs^1^	$42,176 ($61,518)	$39,334 ($61,278)	$36,658 ($51,967)	$33,790 ($50,858)	$26,003 ($29,304)	$147,725 ($323,368)
Total disease-related healthcare costs	$9784 ($16,086)	$12,137 ($20,047)	$4280 ($9,174)	$12,922 ($13,587)	$10,438 ($10,915)	$4048 ($6,838)
Total costs of outpatient prescriptions	$1781($4549)	$1978($4891)	$1499($4073)	$1631($4117)	$1904($5951)	$917($1419)
Emergency room (ER) visits						
Patients with an ER visit	28,047 (25.7%)	11,708 (25.2%)	3261 (9.4%)	11,701 (44.8%)	1364 (99.3%)	10 (8.6%)
Number of ER visits	1.2 (0.6)	1.2 (0.8)	1.1 (0.5)	1.1 (0.4)	1.2 (0.6)	1.0 (0.0)
Total ER Costs^1^	$1768 ($3292)	$1288 ($3531)	$1875 ($3527)	$2117 ($2883)	$2646 ($3300)	$327 ($613)
Outpatient office visits						
Patients with an office visit	79,539 (73.0%)	30,118 (64.8%)	29,120 (83.7%)	19,011 (72.8%)	1150 (83.8%)	111 (95.7%)
Number of office visits	2.7 (2.3)	3.0 (2.5)	2.3 (1.9)	2.9 (2.4)	3.1 (2.5)	3.2 (2.1)
Total outpatient office visit costs^2^	$297 ($340)	$316 ($335)	$268 ($368)	$307 ($302)	$327 ($278)	$406 ($330)
Inpatient admissions						
Patients with an admission	4242 (3.9%)	4069 (8.7%)	87 (0.3%)	80 (0.3%)	6 (0.4%)	0 (0.0%)
Number of admissions	1.0 (0.2)	1.0 (0.2)	1.0 (0.1)	1.0 (0.2)	1.0 (0.0)	0.0 (0.0)
Average length of stay	4.7 (3.7)	4.8 (3.7)	4.5 (2.8)	4.3 (2.8)	7.3 (8.1)	0.0 (0.0)
Total inpatient costs^3^	$29,656 ($33,754)	$29,526 ($33,662)	$40,441 ($43,968)	$25,770 ($23,336)	$13,505 ($4099)	$0 ($0)

Abbreviations: *ER*, Emergency Room; *SD*, Standard Deviation

^1^Average costs of ER visits calculated for just those with at least ER visit

^2^Average costs of outpatient office visits calculated for just those with at least one outpatient office visit

^3^Average costs of inpatient admissions calculated for just those with at least one inpatient admission

Mean healthcare costs were lowest for patients whose index fracture diagnosis was made by an orthopedist ($30,538 ± $53,202). Mean inpatient costs were highest for those diagnosed by an internal medicine physician ($40,489 ± $64,441). Mean outpatient pharmacy costs were lowest for patients whose fragility fracture diagnosis was made by a geriatrician ($3251 ± $4517) and highest for those diagnosed by a family medicine physician ($4108 ± $10,502).

The mean annual disease-related healthcare costs among fragility fracture patients were $9784 (± $16,086), or 22.1% of overall healthcare costs (Table 5). Patients diagnosed in outpatient hospitals or ERs had a higher proportion of disease-related healthcare costs (45.8% and 45.0%), compared with inpatient admissions, outpatient office visits and urgent care (17.0%, 20.5%, 15.3%, respectively).

Mean annual disease-related costs were highest for those diagnosed by a geriatrician ($16,078 ± $27,510) or endocrinologist ($16,397 ± $23,915) and lowest for those diagnosed by an orthopedist ($7690 ± $12,866). Higher costs among those diagnosed by a geriatrician or endocrinologist were driven by the larger proportion of patients with a disease-related inpatient admission (12.8% and 15.9%, respectively) and a larger proportion of patients with a disease-related ER visit (36.2% and 40.9%, respectively).

## Discussion

This claims-based analysis of postmenopausal women with fragility fracture provides insight into the demographic characteristics, clinical conditions, treatment patterns, healthcare costs and utilization during the year following fragility fracture overall and stratified by site of care of fracture diagnosis. It was found that 26.6% of the patients had a subsequent fragility fracture, while rate of osteoporosis treatment and diagnosis was notably low (19.6% with diagnosis and 14.1% with treatment). The inpatient setting was the most common site of care of fragility fracture diagnosis (42.7%), and this cohort was older, sicker (e.g., higher DCI score, higher baseline costs), more likely to have a subsequent fragility fracture, more likely to a severe hip or vertebral type of subsequent fragility fracture, had a higher rate of osteoporosis diagnosis and a higher rate of treatment compared with patients diagnosed with fragility fractures in outpatient sites of care. Women diagnosed with fragility fracture in the inpatient setting incurred the highest healthcare costs ($71,561 ± $84,072) during follow-up which may be attributable to their higher rate of subsequent fractures (26.6%). The lower prevalence of subsequent fractures in the follow-up period might be due to the younger age of patients diagnosed with fragility fracture in the outpatient settings [19]. Hip fractures are among the costliest fracture site and are frequently followed by surgery and lengthy rehabilitation [20, 21]. Results from this study support the need for earlier osteoporosis screening (leading to earlier diagnosis and treatment) to potentially prevent initial and subsequent fractures (particularly those requiring hospitalization) leading to increased burden to both patients and costs to society.

The high rate of subsequent fractures among older women, in general, is supported by several studies of Medicare and commercial populations [22–24]. In a claims analysis among female Medicare beneficiaries 65 years of age and older, 10–31% had a subsequent fracture within 1–5 years [22]. Consistent with our analysis, the majority of these subsequent fractures were non-hip/non-vertebral which emphasizes the need for physical therapy aimed at preventing falls leading to subsequent NHNV fractures. Among older men and women enrolled in Medicare, there was a 2.5 greater risk of fracture within 12 months among those with a history of fracture [23]. Prior hip fracture was identified among 29% of women aged 50 + diagnosed with a hip fracture between 2008 and 2013 with commercial and Medicare Advantage plans [24]. The low osteoporosis treatment rate after fragility fracture diagnosis found in this study is also consistent with prior literature [25–27]. In the current analysis, patients diagnosed with fragility fracture in the inpatient setting had the highest proportion of osteoporosis treatment initiation during the follow-up period; however, it was still only 27.7%. These results are similar to a claims-based study by Solomon et al. using data from 2002 to 2011 which reported that 24.0% of patients diagnosed with a fragility fracture during an inpatient admission were treated with osteoporosis therapy during the 12 months following hospital discharge. In that analysis, it was found that 70% of patients were treated with oral bisphosphonates, 0.3% with denosumab, and 2.6% with teriparatide. Results from the current and more recent analysis show that even among this highest risk cohort (i.e., those diagnosed with fragility fracture during a hospitalization) that treatment rates remain low, and of those who do receive treatment most are prescribed an oral bisphosphonate despite non-oral (and more potent) options available.

In clinical practice, fragility fractures are an indicator of an osteoporosis; however, less than a quarter of individuals with fragility fracture were diagnosed with osteoporosis during the follow-up period and only approximately 10% were diagnosed with osteoporosis during the year prior to fracture [28]. The low rate of osteoporosis diagnosis is likely due to lack of recognition and awareness of the underlying disease (leading to undercoding on healthcare claims). Bone density scans are also indicative of an osteoporosis diagnosis; however, we observed low utilization of these scans as well. A literature review of Canadian practice patterns observed similarly low osteoporosis diagnosis rates among individuals with fragility fracture [29]. This lack of disease awareness contributes to underdiagnosis of osteoporosis that undermines efforts for appropriate treatment [30, 31]. The majority of patients with fragility fracture were, as expected, diagnosed with the initial fracture by an orthopedist. However, few patients (43.3%) with the fragility fracture had subsequent care with their index physician specialty provider. Among those fragility fracture patients in the orthopedics cohort, less than 10% were seen by family medicine or internal medicine and less than 3% were seen by rheumatology and endocrinology specialties. This suggests that patients are not receiving the subsequent care they need for the long-term management of osteoporosis.

There are several strengths to the analyses presented here. First, this study used retrospective claims data, which provides a large, heterogeneous patient population. Unlike clinical trials that are subject to strict inclusion criteria and surveys that are subject to small groups and memory biases, this study of real-world claims captured medication utilization data from a broad group of osteoporosis and fragility fracture patients in clinical practice. It should be noted, however, that this was not a comparative study. Differences in baseline characteristics varied by site of care cohorts and results were not adjusted for baseline differences. Claims studies are subject to several potential limitations. These data were subject to data entry errors or miscoding. Claims data can identify that a medication was dispensed, but not that the medication was administered or taken as prescribed. This analysis was performed among patients with commercial or Medicare Supplemental insurance, and therefore may not be generalizable to those with other insurance types or without insurance coverage. Finally, patients were not necessarily newly diagnosed with fragility fracture in our sample given that a full patient history was not accessible.

## Conclusion

Patients with a fragility fracture had a high rate of subsequent fractures and high cost of care, especially for those requiring hospitalization, so screening and prevention are important to avoid the burden to patients and cost to society. Further, patients diagnosed with fragility fracture in the outpatient settings were younger and had the lowest rate of osteoporosis diagnosis and treatment rates following fracture. Targeting all patients with fragility fracture and particularly those diagnosed in the outpatient setting is of utmost importance for earlier screening, treatment, and fall prevention therapy to potentially avoid hospitalizations and subsequent fractures and to improve patient quality of life. Understanding initial engagement in care, diagnosis, and subsequent sites of care might identify opportunities to decrease subsequent fractures and halt the growing healthcare costs experienced by an aging population.

## Data Availability

The data that support the findings of this study are available from Merative. Restrictions apply to the availability of these data, which were used under license for this study.
